# High-quality genome assembly and multi-omics analysis of pigment synthesis pathway in *Auricularia cornea*

**DOI:** 10.3389/fmicb.2023.1211795

**Published:** 2023-06-15

**Authors:** Xiaoxu Ma, Lixin Lu, Fangjie Yao, Ming Fang, Peng Wang, Jingjing Meng, Kaisheng Shao, Xu Sun, Youmin Zhang

**Affiliations:** ^1^Lab of Genetic Breeding of Edible Fungi, Horticultural, College of Horticulture, Jilin Agricultural University, Changchun, China; ^2^Guizhou Academy of Agricultural Sciences, Guizhou Key Laboratory of Edible Fungi Breeding, Guiyang, China; ^3^Country Engineering Research Centre of Chinese Ministry of Education for Edible and Medicinal Fungi, Jilin Agricultural University, Changchun, China; ^4^Economic Plants Research Insitute, Jilin Academy of Agricultural Sciences, Changchun, China

**Keywords:** *Auricularia cornea*, genome, transcriptome, metabolome, shikimate pathway, GDHB pigment

## Abstract

Owing to its great market potential for food and health care, white *Auricularia cornea*, a rare edible fungus, has received increased attention in recent years. This study presents a high-quality genome assembly of *A. cornea* and multi-omics analysis of its pigment synthesis pathway. Continuous Long Reads libraries, combined with Hi-C-assisted assembly were used to assemble of white *A. cornea*. Based on this data, we analyzed the transcriptome and metabolome of purple and white strains during the mycelium, primordium, and fruiting body stages. Finally, we obtained the genome of *A.cornea* assembled from 13 clusters. Comparative and evolutionary analysis suggests that *A.cornea* is more closely related to *Auricularia subglabra* than to *Auricularia heimuer*. The divergence of white/purple *A.cornea* occurred approximately 40,000 years ago, and there were numerous inversions and translocations between homologous regions of the two genomes. Purple strain synthesized pigment via the shikimate pathway. The pigment in the fruiting body of *A. cornea* was γ-glutaminyl-3,4-dihydroxy-benzoate. During pigment synthesis, α-D-glucose-1P, citrate, 2-Oxoglutarate, and glutamate were four important intermediate metabolites, whereas polyphenol oxidase and other 20 enzyme genes were the key enzymes. This study sheds light on the genetic blueprint and evolutionary history of the white *A.cornea* genome, revealing the mechanism of pigment synthesis in *A.cornea*. It has important theoretical and practical implications for understanding the evolution of basidiomycetes, molecular breeding of white *A.cornea*, and deciphering the genetic regulations of edible fungi. Additionally, it provides valuable insights for the study of phenotypic traits in other edible fungi.

## Introduction

*Auricularia cornea*, which belongs to the genus *Auricularia* Bull. ex Juss. (Basidiomycota), is a highly nutritious, medicinally valuable fungus that can be used as both medicine and food (Baldrian and Lindahl, 2011). The rare white variety has great market potential due to its popularity. Genomic information is the basis for studying *A. cornea* color inheritance. It is also an important resource for studies including gene mapping, genetic diversity analysis, classification and phylogeny, germplasm evaluation, and molecular marker-assisted breeding (Wu et al., 2015; Cao et al., 2016). However, while the lack of a chromosome-level genome significantly limits *A. cornea* research and development, it also causes low integrity in the assembly of genome sequencing results used for research, limits polymorphic sites, and fails to reveal all genetic information characteristics. Therefore, insights into the basic genome structure of *A. cornea* are required to obtain a chromosome-level genome.

Over 30 edible fungi, including *Auricularia heimuer*, *Gloeostereum incarnatum*, *Agaricus bisporus*, *Flammulina velutipes*, *Pleurotus ostreatus*, and *Ganoderma lucidum*, have had their genomes sequenced and assembled, thanks to advances in sequencing techniques. These reference genomes are now important resources for studies regarding molecular marker-assisted breeding, population genetics, and comparative genomes (Morin et al., 2012; Young-Jin et al., 2014; Zhu et al., 2015; Qu et al., 2016; Yuan et al., 2019; Fang et al., 2020; Jiang et al., 2021). Although *A. cornea* sequencing and assembly have been completed, having a 78.50 M genome size and 51 contigs (Dai et al., 2019). Notably, current second- and third-generation sequencing methods can only assemble the genome to a contig/scaffold level, but the Hi-C method extends the draft genome. The latter involves sequencing DNA fragments after cross-linking and enrichment based on linear distances and close spatial structures. The analyzed sequencing data can reveal the interactions between DNA segments, deduce the genome 3D spatial structure, and determine the possible regulation between genes in order to construct a genome close to the chromosomal level. In addition to acquiring a chromosome-level genome, Hi-C-assisted assembly can improve the quality and continuity of the assembled genome by error correction, further optimizing the assembly results by determining whether the genome contains redundancy. As for giga-genome and polyploidy species, Hi-C can achieve effective mounting and haplotype analysis to produce a high-quality reference genome (Zhang et al., 2019).

Except for the high-quality genome analysis, the end products of the cellular regulatory process, known as metabolites, can sufficiently explain the phenotypic changes of a biological system but inadequately analyze metabolite diversity and the genetic mechanism. Therefore, synergistic analysis based on transcriptomics and metabolomics can provide accurate information regarding the gene–metabolite interaction and build a regulatory network for corresponding metabolites, thereby facilitating the study of gene function and metabolic pathways. The association analysis of transcriptome and metabolome was extensively used to elaborate the genetic and regulatory mechanisms of plants’ metabolites (Cho et al., 2016; Hu et al., 2016; Wang et al., 2017; Li et al., 2018; Tingting et al., 2019). It has only been published in a few studies involving edible fungi, such as the anti-browning mechanism in *A. bisporus*, β-glucoside inhibitor in increasing cold-resistance of *Volvaria volvacea*, and high-temperature stress on *Lentinus edodes* (Zhao et al., 2019; Cai et al., 2021; Gong et al., 2022).

The color of *A. cornea*’s purple fruiting body varies from dark to light, showing a series of continuous changes. This indicates that the purple fruiting body of *A. cornea* is regulated by multiple genes in the pigment synthesis process, which presents a quantitative trait with continuous change. The white strain’s fruiting body is completely white, which could be caused by the deletion or mutation of one or more key enzyme genes in the pigment synthesis pathway, which blocks the entire pathway and results in no pigment synthesis. A previous study demonstrated that the key enzyme influencing pigment synthesis in *A. cornea* was identified as glutamine-dependent amidotransferase (Gn-AT), which was also hypothesized to be the key enzyme for the synthesis of the pigment γ-glutamine-4-hydroxy-benzoate (GHB) (Wang et al., 2019). However, the complete pigment synthesis pathway, major genes involved in this pathway, and melanin type contained in *A. cornea* pigment remain unclear.

In this study, two sequencing models (Continuous Long Reads (CCS) Library and Circular Consensus Sequencing (CCS) Library) combined with Hi-C-assisted assembly were used to perform high-throughput sequencing on the white *A. cornea* genome to assemble the genome at the chromosome level. At various growth stages, the transcriptome and metabolome of the white strain ACW001 and purple strain ACP004 were compared and analyzed using a high-quality reference genome. Through multi-omics association, the coexpression of differentially expressed genes and metabolites was effectively analyzed in order to provide accurate information and construct a pigment synthesis model for the interaction between pigment synthesis genes and metabolites. Understanding the pathway and mechanism of pigment synthesis and its varieties can lay a solid foundation for the efficient breeding of white *A. cornea* and for the color genetic study of other edible fungi.

## Results and analysis

### Genome sequencing and Hi-C-assisted assembly

**Figure 1:**
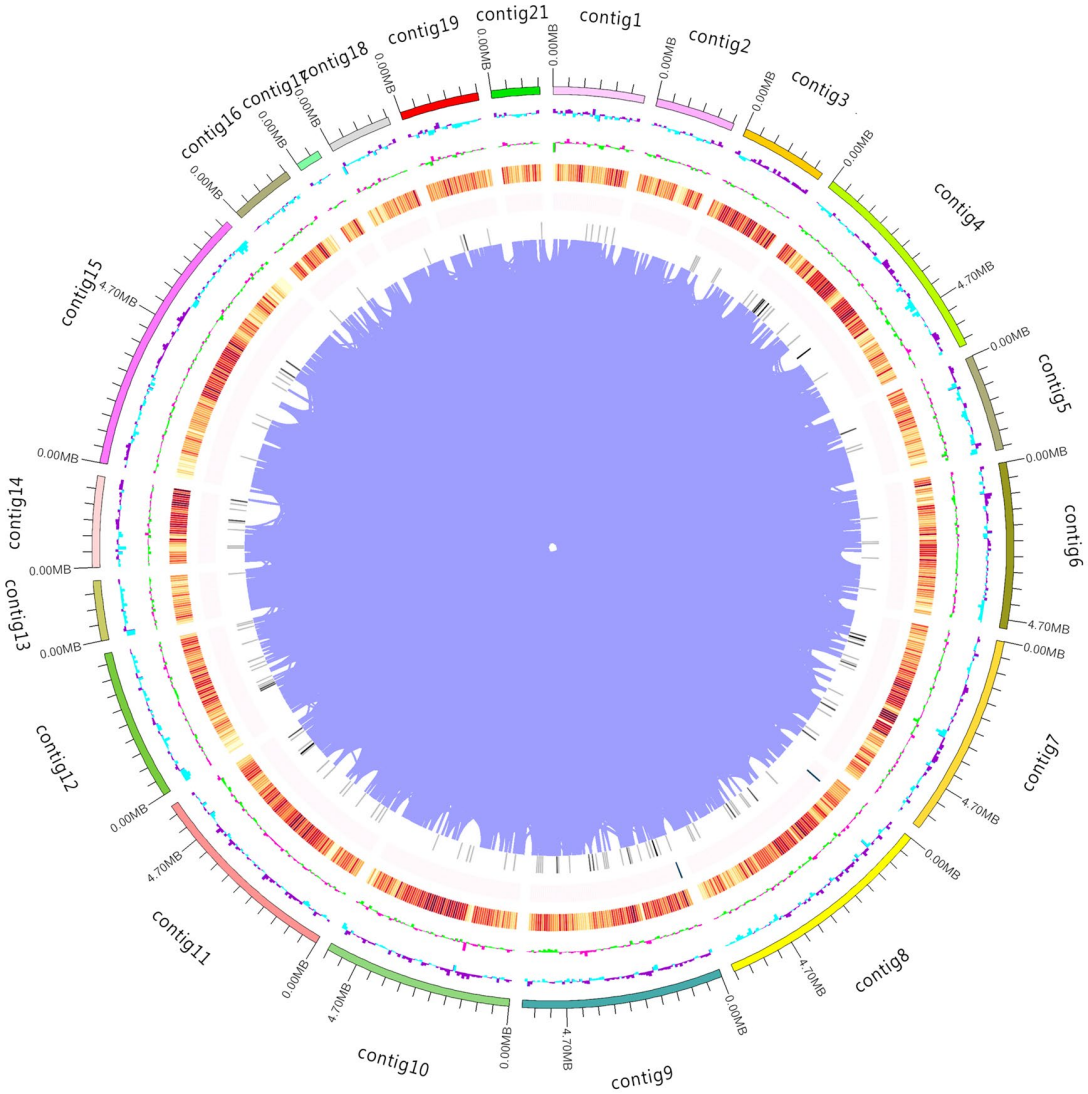
Whole genome map. The outermost circle represents the genomic sequence position coordinates. Moving from outer to inner, the next circles represent the genomic GC content, GC skew value, gene density (coding genes, rRNA, snRNA, and tRNA densities are counted separately, with darker colors indicating higher gene density in the window), and chromosome duplication.

16 Gb raw data were generated from PacBio Sequel and Illumina NovaSeq PE150. After selection, assembly, and optimization, 79.01 Mb genome sequences comprising 28 contigs were obtained (Figure 1). Hi-C captured 24,808,063 pairs of reads that could pair with the genome, 17,376,358 valid read pairs were obtained after HiCUP quality control, and the reads were aligned to 23 contigs. Because the intra-chromosome interaction probability markedly exceeded that of inter-chromosome, different contigs were divided into different chromosomes, and 23 contigs were clustered into clusters resembling chromosomes. 99.63% of assembled sequences were mounted to 13 clusters; the N50 value was 6.03 Mb and N90 value was 4.56 Mb (Table 1). On the same chromosome, the interaction probability decreased as the interaction distance increased and the contigs could be ordered and oriented. In addition, a collinearity analysis of the existing genetic linkage and physical maps was performed (Supplementary Figure S1). BUSCO evaluation showed that only 26 of 1,764 single-copy genes were missing, with a 98.53% assembly integrity, exceeding the 97.60% of the Basidiomycota database and 98.30% of the fungal database (Manni et al., 2021a,b). The assembled genome is larger than *A. heimuer* (49.76 Mb) and *Auricularia polytricha* (38.69 Mb) of *Auricularia*, and similar to *Auricularia subglabra* (74.92 Mb). The current *A. cornea* genome had 23 fewer contigs than the previously disclosed genome, with higher N50 and N90 values and comparable guanine–cytosine (GC) content, suggesting that the current genome has higher quality and completeness.

**Table 1:** Assembly results of CLR and CCS.

Assembly feature	CCS Library	CLR Library
Assembly size (Mb)	79.01	78.24
Number of contigs	28	70
Contig N50 (bp)	5,662,720	23,93,831
Longest contig (bp)	8,179,760	5,514,487
Number of clusters	13	13
Assembly of genome (%)	99.63	74.35
GC (%)	59.06	59.65
Repeat region of assembly (%)	5.74	5.17
Number of predicted gene models	18,574	19,133
Average gene length (bp)	1,310	1,274

### Genome annotation

A total of 28,142 repetitive sequences were predicted, with a combined length of 4.75 megabases (Mb), accounting for 5.74% of the genome. Numerous transposable elements were identified, with long terminal repeat sequences (LTRs) being the most abundant, occupying 2.49% (2.06 Mb) of the genome, followed by long interspersed nuclear elements (LINEs), which accounted for 0.25% (0.21 Mb) of the genome. The proportion of LTRs in white *A. cornea* was significantly different from that in purple *A. cornea* (10.33%). These differences may be due to natural selection, species lifestyle, and ecological niche (Lan and Constabel, 2011), and could be an important factor causing the variations between white and purple *A. cornea*. A total of 9,423 simple sequence repeat (SSR) loci were detected, with a similar SSR locus density to black wood ear. The number of 3-base repeat units was the highest, which was 4,677, representing 49.63% of the total SSR repeat units.

To avoid the impact of repetitive sequences on the quality of gene prediction, we masked these regions during gene prediction. Using the Augustus program, we predicted a total of 18,574 coding genes, with a combined length of 24.33 Mb and an average length of 1,310 (bp). The coding regions accounted for 29.41% of the entire genome. Of these genes, 14,616 (78.69%) were supported by transcripts with a coverage of >80%. To gain a better understanding of the functions of the predicted genes, we compared them against eight widely used functional databases. Our results showed that 15,933 (86.20%) of the predicted genes had putative functions in these databases. Specifically, 14,844 (79.92%) had homologs in the Non-Redundant Protein Database (Nr), 9,278 (49.95%) were known proteins in the Gene Ontology (GO) database, 14,486 (77.99%) were known proteins in the Kyoto Encyclopedia of Genes and Genomes (KEGG), 1,929 (10.39%) were known proteins in the Cluster of Orthologous Groups of proteins (KOG) database, 2,763 (14.88%) were known proteins in the SwissProt database, 9,278 (49.95%) were known proteins in the Pfam database, 809 (4.36%)were known proteins in the Carbohydrate-active enzymes (CAZy)database, contained 893 CAZyme modules, and 475 (2.56%) were known proteins in the Transporter Classification Database (TCDB). Furthermore, we identified a total of 2.36 Mb of non-coding RNAs (ncRNAs), which accounted for 2.99% of the assembled genome. Among them, 211 were transfer RNAs (tRNAs), and 906 were ribosomal RNAs (rRNAs).

### Secondary metabolic gene cluster analysis

The *A. cornea* genome contains 32 secondary metabolic gene clusters, including 20 terpene synthases, 1 type I polyketide synthase (PKS), 2 indole synthases, 1 nonribosomal peptide synthase, and 8 nonribosomal peptide synthases-like. While the most prevalent secondary metabolites of fungi are polyketides, a group of compounds synthesized by PKSs, quinones are common polyketide fungal pigments produced via the polyketide pathway (Feng et al., 2015). Therefore, type I PKS significantly contributes to fungal polyketide synthesis, and its gene cluster contains 9 genes located on contig 15.

### Comparison and evolutionary analysis

**Figure 2:**
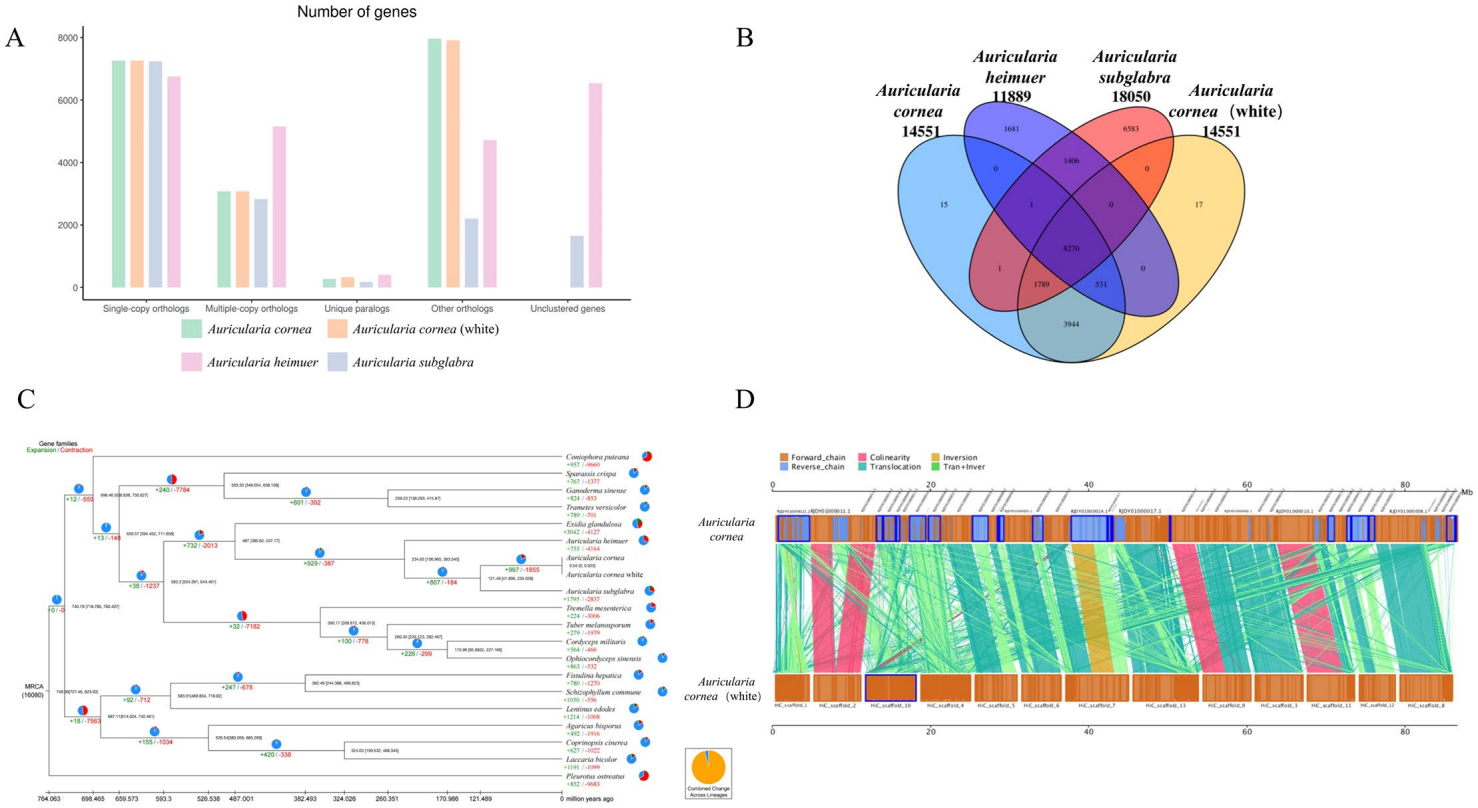
Genomic analyses. **(A)** Orthologous genes in the genomes of *A. cornea*, *A. cornea* white, *A. subglabra* and *A. heimuer*. **(B)** Unique and shared gene families among the four genomes. **(C)** Phylogenetic tree of 20 edible fungi. **(D)** The genome collinearity analysis of the *A. cornea*.

To study the evolution of the gene family of white *A. cornea*, we compared the protein sequences with three other *Auricularia* species (Purple *A. cornea*, *A. heimuer* and *A. subglabra*) and 16 other edible fungi. A total of 1,997 homologous gene families were conserved in all compared genomes (Figure 2A). In the four *Auricularia* species genomes, there were approximately 8,270 conserved homologous gene families. Furthermore, we found 15 and 17 specific homologous gene families for white/purple *A. cornea*, respectively (Figure 2B). Functional analysis indicated that these specific genes are associated with carbon source degradation and secondary metabolite metabolism. We then performed phylogenetic evolutionary analysis of 641 conserved single-copy homologous genes in all edible mushrooms, and the phylogenetic tree of 19 species showed that the *Auriculariales* clustered together on the same branch. *A. cornea* was closer to *A. subglabra* than to *A. heimuer* in terms of genetics (Figure 2C), which is consistent with previous reports. These results indicate that our phylogenetic tree accurately reflects the evolutionary relationship. The divergence time between *Auriculariales* and other orders was about 487 (390.62–537.77) Mya, and the divergence time between *A. cornea* and *A. subglabra* within the *Auriculariales* was about 121.49 (41.90–235.03) Mya. Based on the divergence time of white/purple *A. cornea*, it can be inferred that about 40,000 years ago, the purple *A. cornea* may have been affected by environmental factors or natural mutations that damaged the pigment synthesis pathway, resulting in the white mutant.

In the evolutionary process of *A. cornea*, contraction of gene families is more common than expansion, and a total of 997 gene families have been expanded in *A. cornea*. The number of contracted gene families in *A. cornea* (1,855) is much smaller than in the same genus *A. heimuer* (4,164) and *A. subglabra* (2,837). Functional analysis shows that these contracted gene families are mainly related to degradation metabolism, immune system, sorting, and degradation. These genes play a critical role in adapting to harsh environments and substrate degradation, leading to the widespread distribution of *A. cornea* in *Auricularia*.

We conducted a whole-genome collinearity analysis between *A. cornea* and purple *A. cornea* and found numerous inversions and translocations between homologous regions of the two genomes (Figure 2D). For instance, we observed a translocation between scaffold 7 of white *A. cornea* and contig14 of purple *A. cornea*, as well as an inversion and translocation between scaffold 1 of white *A. cornea* and contig21 of purple *A. cornea*. However, only a few regions showed highly conserved syntenic blocks shared between the two genomes, such as scaffold 2 of white *A. cornea* and contig11 and contig13 of purple *A. cornea*. These findings suggest that a series of chromosome fusions or breakages may have occurred during the long evolutionary history of *A. cornea*, resulting in the observed genomic diversity. This genomic diversity may play a crucial role in various aspects of the organism’s biology, including morphological formation, lifestyle, and environmental adaptation.

### The pigment of *Auricularia cornea* is synthesized by shikimate pathway

**Figure 3:**
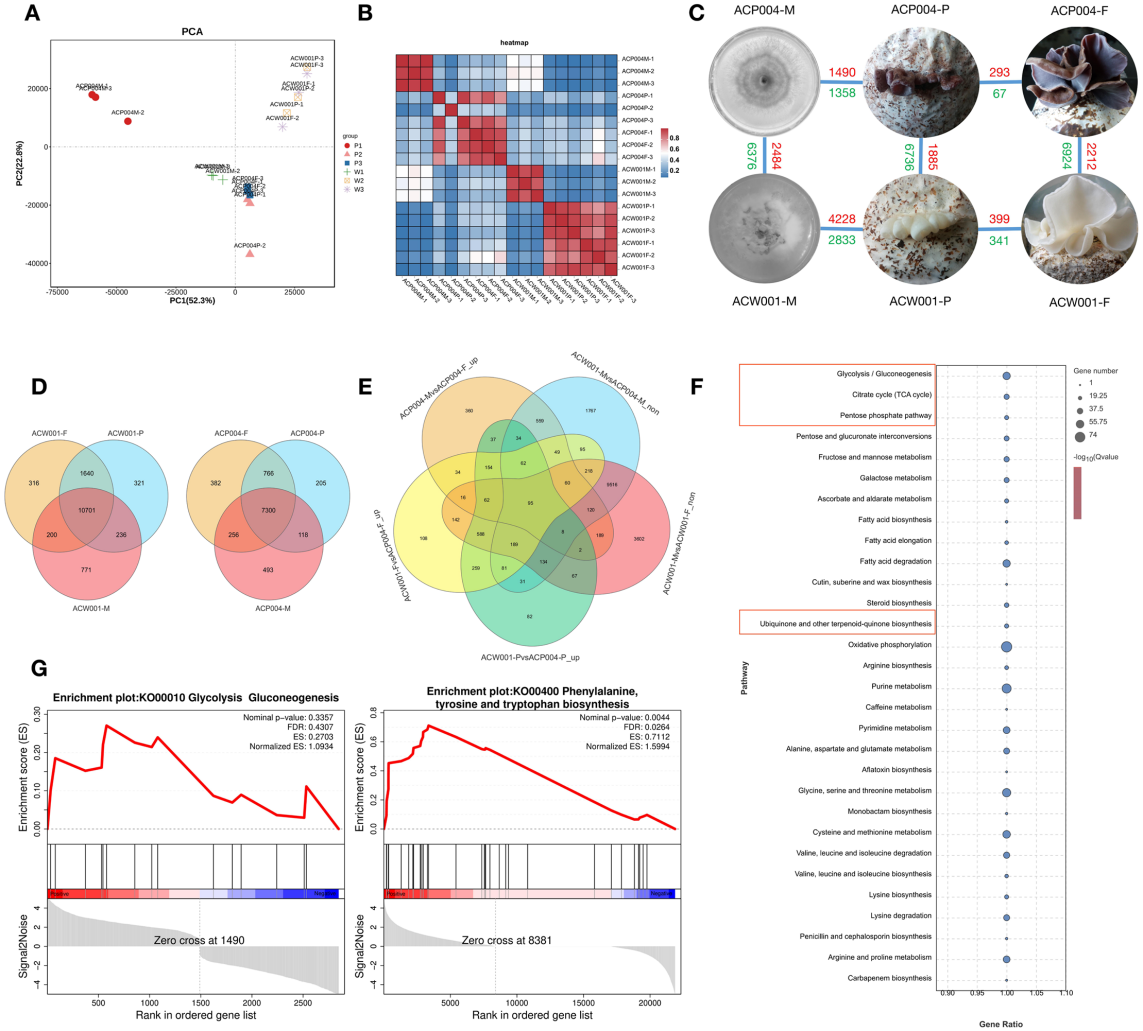
Transcriptome-based analysis of genes involved in pigment synthesis. **(A)** PCA analysis, the more similar the sample composition is, the closer the distance in the PCA plot. **(B)** Correlation heat map: repeatability between repeated samples can be investigated. **(C)** DEG distribution in each sample, red represents upregulation and green represents downregulation. **(D)** Venn diagram of differential genes during the three development periods of purple and white strains. **(E)** Venn diagram of targeted genes. **(F)** KEGG enrichment bubble diagram. The red boxes represent the pathways associated with predicted pigment synthesis. **(G)** GESA enrichment ES curves. The gene enriched when ES reached the highest score is the major gene of the pathway.

Color is an important agronomic trait of *A. conrea*. In order to clarify the regulation gene and pathway of pigment synthesis, we conducted an RNA-seq experiment on the mycelium period (Mycelium as control treatment: WCK and PCK), primordium period (The 8th day of fruiting: W08 and P08), and fruiting body period (The 15th day of fruiting: W15 and P15) of white strains ACW001(W) and purple strains ACP004(P) based on the ACW001-33 genome. Pigment synthesis-related regulatory genes were selected by combining phenotypic differences with gene expression levels. Based on expression information, principal component analysis (PCA) was performed (Figure 3A), and we calculated the Pearson correlation coefficient of every two samples (Figure 3B), excluding two samples with low repeatability, and DEGs in purple and white strains were then analyzed (Figures 3C,D).

Morphological observation of *A. cornea* at different stages revealed that pigment secretion began in the primordium period. From the mycelium to fruiting body period, purple parents underwent a color change, whereas white parents remained unaltered. Therefore, we selected the intersection of upregulated DEGs in W08 *VS* P08, W15 *VS* P15, and PCK *VS* P15 and non-DEGs in WCK *VS* PCK and WCK *VS* W15 to perform KEGG annotation analysis (Figure 3E). The enrichment analysis revealed that the pathways of ubiquinone and other terpenoid-quinone biosynthesis, glycolysis, pentose phosphate, and TCA cycle were significantly enriched (Figure 3F). Another gene set enrichment analysis (GSEA) performed using the two sample gene sets of PCK *VS* P08 and PCK *VS* P15 from the purple strains. The pathways of glycolysis, phenylalanine, tyrosine, and tryptophan were significantly upregulated during purple strain development (Figure 3G). Eleven core genes enriched by GSEA included phosphate synthase, farnesyl pyrophosphate synthase, polyphenol oxidases (PPO: PPO1, PPO2, and TYPR), glutamic oxaloacetate aminotransferase (GOT), aromatic amino acid aminotransferases (AMT and ARO), alcohol dehydrogenases (TDH and ADHT), and aldolase A (ALDA). It was found that the pigment synthesis pathway related to KEGG enrichment and GSEA analysis results is the Shikimate pathway, Shikimate was synthesized via glycolysis and the pentose phosphate pathway, and the enriched core genes were also related to these pathways. Therefore, *A. cornea* pigment was preliminarily inferred to be synthesized via the shikimate pathway.

### The pigment of *Auricularia cornea* is composed of GDHB

**Figure 4:**
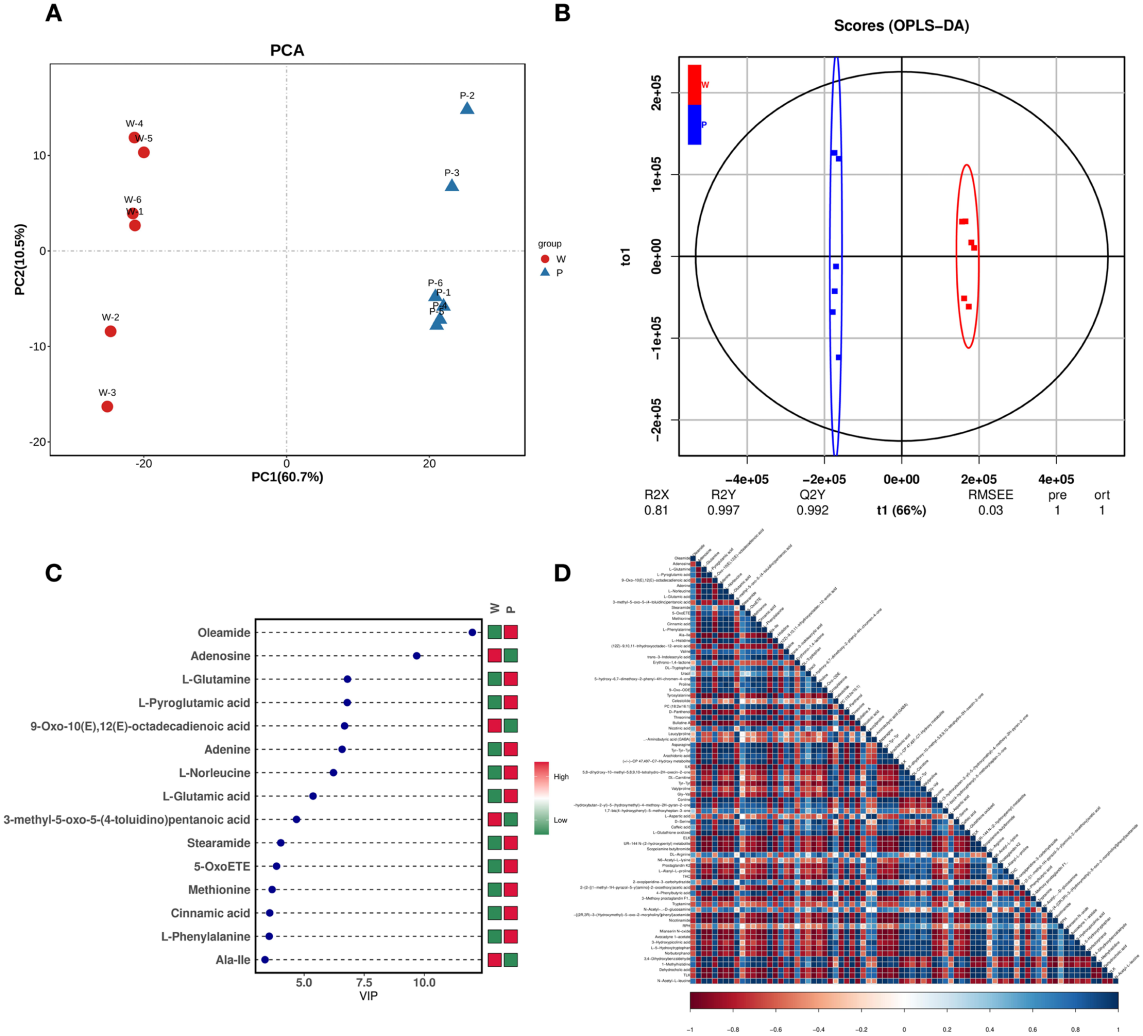
Metabolome-based analysis of metabolites associated with pigment synthesis. **(A)** PCA analysis, PC1 describes the most obvious features in the multidimensional data matrix, and PC2 describes the most significant features in the data matrix except PC1. **(B)** OPLS-DA, use the classification effect of R2X, R2Y, Q2 models. **(C)** DEM VIP map, color on the right side indicates the abundance of each metabolite in different groups, red represents upregulated, and green represents downregulated. The higher the VIP values, the more they contribute to distinguishing samples. The metabolites with VIP exceeding 1 have significant differences. **(D)** DEM heat map, each row and column represents the sample, and the darker the color, the stronger the correlation between the two samples.

The shikimate pathway synthesizes various pigments, with PPO participating in all pigment syntheses of this pathway, suggesting that transcriptome analysis cannot reveal the specific PPO participation in melanin synthesis. Therefore, we used nontargeted LC–MS to identify the fruiting body metabolites of ACW001 and ACP004, obtaining 899 and 715 metabolites in the positive and negative ion modes, respectively. Under the two ion modes, PCA analysis and the OPLS-DA score plot demonstrated excellent separation effects, effectively differentiating the white and purple strains (Figures 4A,B). The prediction values of the OPLS-DA analysis exceeded 0.9, indicating a reliable result. According to the screening criteria (variable importance in projection [VIP] ≥ 1 and T-test *p* < 0.05), 81 (39 upregulated and 42 downregulated) and 69 (47 upregulated and 22 downregulated) differential metabolites (DEMs) were identified under positive and negative ion modes, respectively (Supplementary Tables S1, S2). When plotted on the VIP chart (Figure 4C) of OPLS-DA, the VIP data corresponding to the DEMs (MS2 levels) in the top 15 differential VIP values in each comparison group showed that glutamate and glutamine in the purple strains were significantly upregulated. Malate, citrate, 2-Oxoglutarate, tyrosine, alanine, and phenylalanine changed synergistically with glutamate and glutamine, as determined by the Pearson correlation coefficients of all DEMs in pairs (Figure 4D). Notably, these metabolites are important in the TCA cycle and shikimate pathway, the TCA cycle generates glutamine, which is a precursor substance for the synthesis of γ-glutaminyl-3,4-dihydroxy-benzoate (GDHB) pigments. The GDHB pigment synthesis pathway is a branch of the shikimate pathway. Many compounds involved in the GDHB pigment synthesis pathway carry glutamine groups, and no other melanin precursor substances have been found. Therefore, it is inferred that *A. cornea* pigment is a single type of pigment synthesized by the GDHB pathway.

### Transcriptome and metabolome association analysis of pigment synthesis pathway

**Figure 5:**
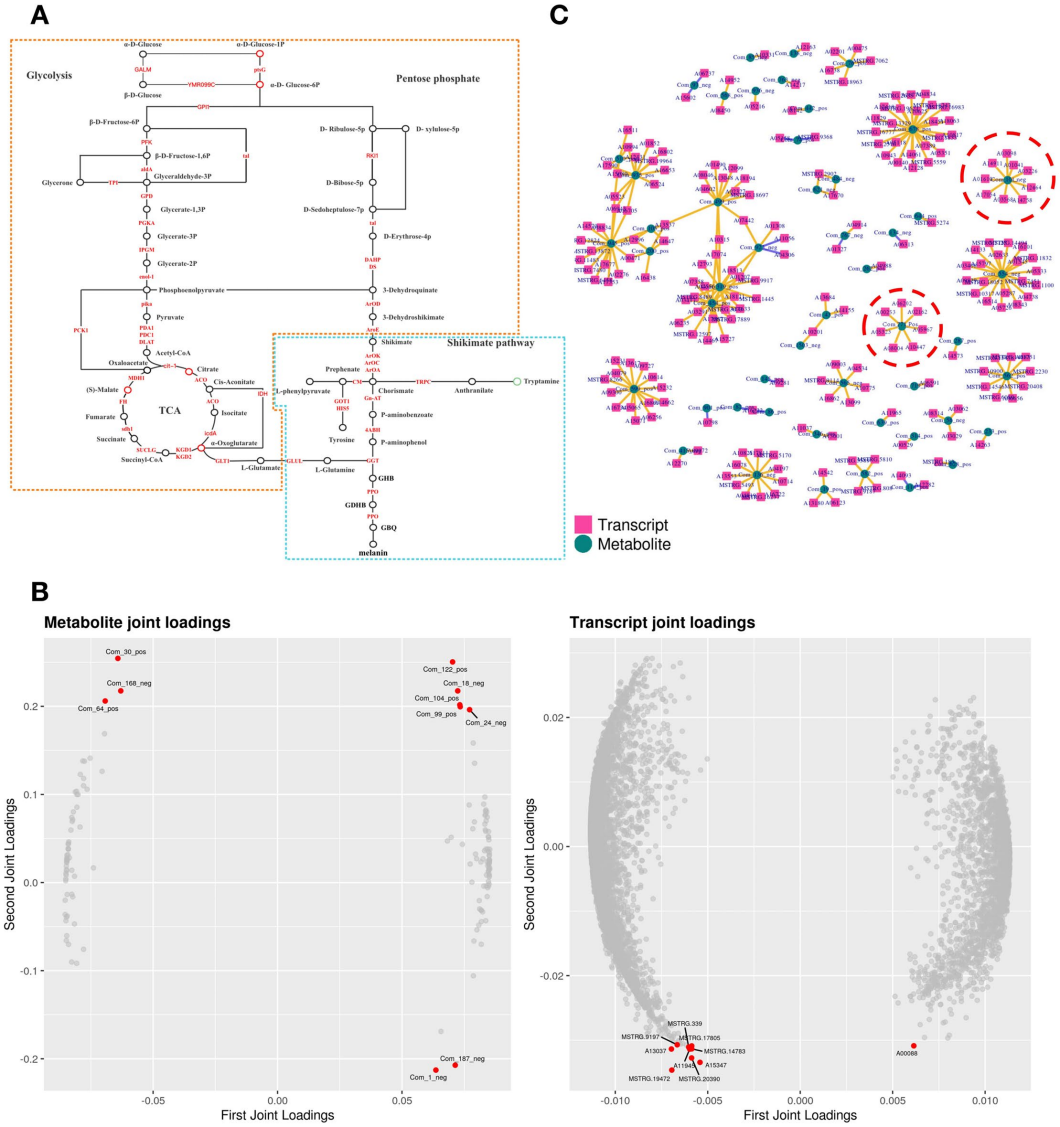
Conjoint analysis model of transcriptome and metabolome. **(A)** Pathway analysis of pigment synthesis. The yellow part is the shikimate pathway, and the blue part is the GDHB pigment synthesis pathway. **(B)** O2PLS loading diagram model. The left side is the metabolomic loading map, and the right side is the transcriptomic loading map. **(C)** Pearson correlation coefficient model. Inside red circles are DEMs and DEGs related to pigment synthesis.

Transcription and metabolism do not happen independently in the biological system, we performed KEGG pathway model analyze related to pigment synthesis based on gene expression level and metabolite abundance to further explain the regulatory mechanism between gene expression and metabolism. It was significantly enriched with glycolysis and the shikimate pathway (*p* < 0.05), indicating a strong consistency between transcriptome and metabolome results. The metabolic biomarker changes associated with shikimate synthesis and metabolism are shown in the network chart (Figure 5A, Orange). Purple strains synthesized phosphoenolpyruvate (PEP) and D-erythrose-4P via glycolysis and the pentose phosphate pathway, followed by shikimate polymerization with the action of DAHP synthase, and pigment synthesis via the shikimate pathway.

### Key metabolites and gene screening in pigment synthesis pathway

Data of gene expression level and metabolite abundance were used to build the O2PLS (bidirectional orthogonal projections to latent structures) loading diagram model (Figure 5B). Citrate (Com_18_neg) and 2-Oxoglutarate (Com_24_neg) in the TCA cycle were positively correlated with A02009 (Aldolase A, ALDA). ALDA participated in the glycolytic pathway; β-D-fructose-1,6P generated glyceraldehyde-3P and finally synthesized acetyl-CoA. ALDA was also implicated in the pigment synthesis pathway in the transcriptome GSEA analysis. Then, construct the correlation coefficient model based on gene expression level and metabolite abundance: After calculating the Pearson correlation coefficients of gene expression level and metabolite abundance, the data of the top 250 DEGs and DEMs with absolute values of correlation coefficients exceeding 0.99 were selected to draw a network diagram (Figure 5C). 2 DEMs (α-D-glucose-1P and glutamate) involving 16 DEGs (Table 2) were closely associated with pigment synthesis. These DEMs and DEGs were responsible for central regulation in pigment synthesis.

**Table 2:** Statistics of genes related to pigment synthesis.

Gene ID	EC number	Symbol	Gene ID	EC number	Symbol	Gene ID	EC number	Symbol
A01041	5.1.3.15	YMR099C	A01610	4.1.1.49	PCK1	A03568	2.6.1.57	ARO1
A03098	5.1.3.3	GALM	A06202	1.1.1.41	IDH1	A17054	2.6.1.9	ARO8
A03226	4.1.2.13	fba	A02162	1.1.1.42	icdA	A05690	1.14.18.1	PPO1
A12464	4.2.1.11	Enol-1	A00253	1.2.4.2	KGD1	A12376	1.14.18.1	PPO2
A14758	2.7.1.40	pkiA	A05967	2.3.1.61	KGD2	A01747	1.14.18.1	TYRP
A14911	2.3.1.12	Dlat	A05323	1.1.1.37	MDH1	A02009	1.2.1.3	ALDA
A10447	1.4.1.14	GOGAT	A08004	1.3.5.1	sdh1			

### Key gene validation in the pigment synthesis pathway

**Figure 6:**
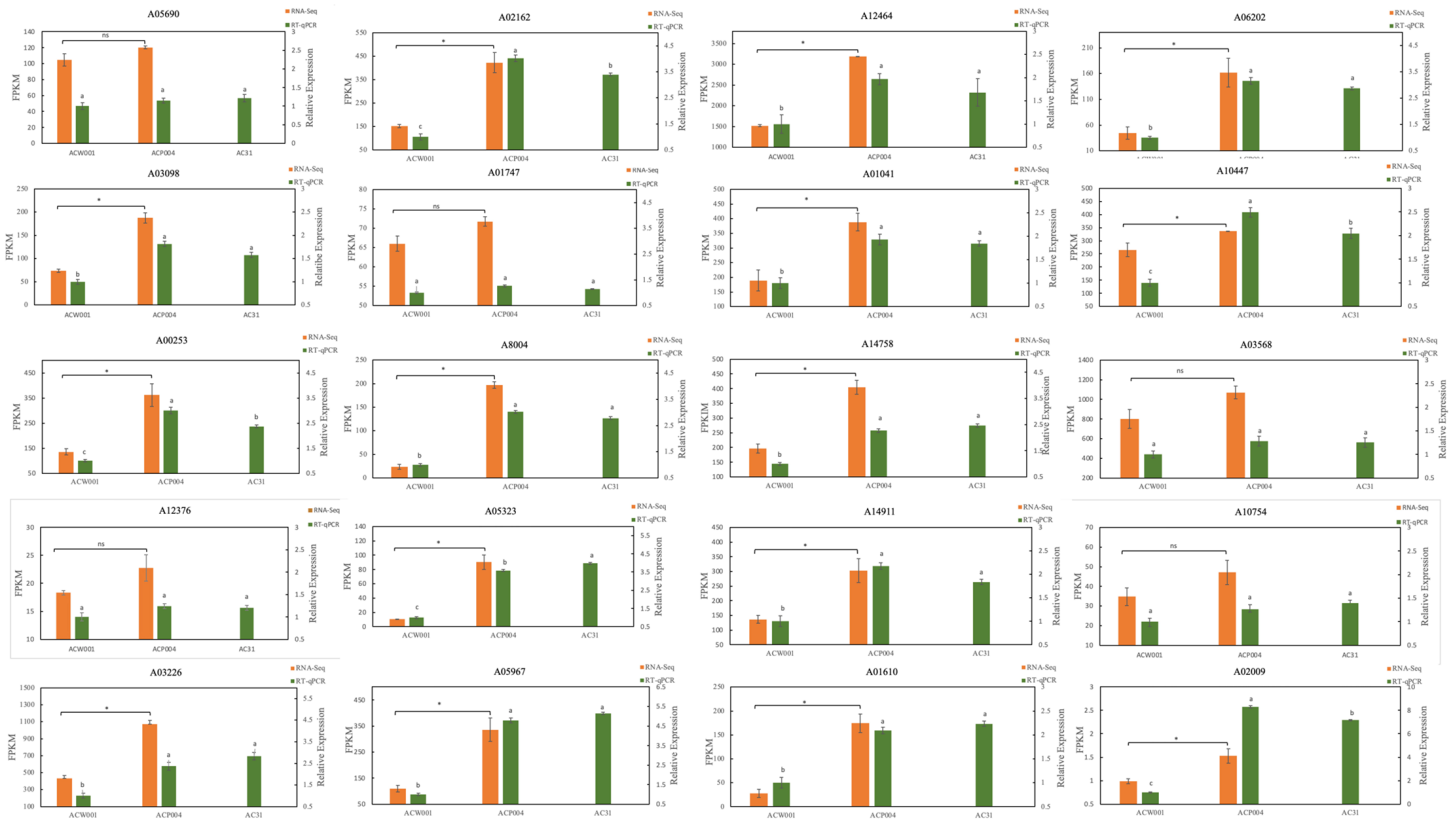
RT-qPCR map of 20 pigment synthesis-related genes. ACW001 is the reference strain. The 20 genes included 17 candidate genes and 3 PPOs.

The strain AC31 (Obtained by monosporal hybridization between ACW001 and ACP004, the color of the fruiting body is pink), white parent ACW001, and purple parent ACP004 were selected for the RT-qPCR analysis of the 17 putative genes (Figure 6, The primer sequence is shown in Supplementary Table S3). The results showed that 15 of the 17 pigment synthesis-associated genes were significantly more expressed in the purple and pink strains than in the white strains. Genes Aromatase1 (ARO1:A03568), ARO8 (A10754) had high expression levels in all strains, without a significant difference. AROs ficantly enriched in GSEA, indicating their important roles in the pathway, despite the absence of a significant expression level differential between the different color strains. The RT-qPCR results demonstrated the accuracy of the association analysis.

## Discussion

### Effects of different sequencing modes on genome assembly

Although both the CLR and CCS libraries can produce high-quality draft genomes, their assembly integrity and accuracy differ significantly. As seen in Table 1, the genomic sizes under these two modes are similar, but the CCS genome contains only 28 contigs. We also observed that CCS assembled more precisely based on the size of contig N50 and the longest contig. The two modes had similar GC content, indicating a high-accuracy sequence. In addition, the Hi-C-assisted assembly exhibited significant differences. Although both modes anchored the genome sequences to 13 clusters, CLR sequence anchoring accounted for only 74.35%, with 21 of 70 contigs engaging in clustering. In contrast, the CCS mode reached 99.63% on anchoring, with 23 of the 28 contigs engaging in clustering. Notably, when performing clustering, one contig appeared in two clusters in the CLR genome, indicating an obvious assembly error. The two modes were similar in repetitive sequences and gene prediction. Overall, CCS obviously outperforms CLR in the genomic sequencing of *A. cornea.* CCS has been widely used in plant and animal genome sequencing but not yet in edible fungi (Rhein, 2021). While the *A. cornea* genome results demonstrated that CCS is suitable for edible fungi and offers greater benefits, they also provided a high-quality genome reference for *A. cornea* and insights into the whole-genome sequencing of other edible fungi.

### The importance of GSEA analysis

Transcriptome analysis revealed that the detection rate of all ACP004 samples was approximately 50% relative to the reference genome, but that of all ACW001 samples was approximately 70%, indicating that ACP004 differed significantly from the reference genome in terms of gene composition and sequences. Notably, GO and KEGG enrichment analyses of gene expression differences may miss some color regulatory genes, as the color difference is likeliest due to changes in gene sequences rather than gene expression levels. Therefore, the search for target genes in this study was based on GSEA analysis, which analyzed the gene sets at different periods of purple parents with color changes. GSEA is not limited to DEGs, as it can also include genes that are easily missed in GO/KEGG but have important biological meaning from the perspective of gene set enrichment (Subramanian et al., 2005, 2007; Tarca et al., 2013). This makes our analysis results more accurate.

### Prediction of pigment synthesis pathways

The GDHB pathway is a branch of the shikimate pathway. For instance, chorismate is synthesized via the shikimate pathway, forming para-aminobenzoic acid under Gn-AT catalyzation (Massière and Badet-Denisot, 1998), which is then oxidized and decarboxylated by 4ABH to yield aminophenol (Weijn et al., 2013). Aminophenol and glutamine under GGT catalyzation yield GHB (Jolivet et al., 1999), which is then oxidized by PPO to form GDHB and re-oxidized by PPO to form stable glutaminy-benzoquinone (GBQ), which finally polymerizes melanin (Figure 5A Blue). We used ultraviolet spectrophotometer and infrared spectroscopy scanning to extract and purify the pigment from *A. cornea* in the early stage, and it was found that the main compound group composed of *A. cornea* pigment is pyrocatechol (Fan et al., 2019). This is consistent with the chemical group composition of GDHB, which also indicates that our analysis results are accurate. The type of pigment in *A. cornea* is the same as that in *A. bisporus* (Hans and Dora, 1981), and GHB and GDHB are also present in other types of edible fungi (Mcmorris and Turner, 1983). Therefore, GDHB may be a natural precursor of melanin in many other species of the Basidiomycotina, whose main phenolic oxidase is PPO (Bell and Wheeler, 1986). Of course, all genes in the pigment synthesis pathway are important, and any mutation or deletion of genes may interrupt pigment synthesis (Wang et al., 2019). Subsequent experiments can change the color of fruiting bodies by inhibiting key genes with higher expression levels.

### PPOs and are key enzyme genes in the pigment synthesis pathway

Three types of PPOs have been found in *A. cornea*, namely PPO1 (A05690), PPO2 (A12376) and TYPR (A01747). PPO is essential for pigment synthesis in animals, plants, and fungi because it can catalyze monophenolase hydroxylation and oxidize o-diphenol to o-quinone; quinones are subsequently oxidized to derivatives. O-quinone and other quinones, amino acids, and proteins form pigments through nonenzymatic polymerization (Lan and Constabel, 2011; Aleksandar et al., 2015). PPO is also a key enzyme in the GDHB pigment synthesis pathway that oxidizes GHB to GDHB and GBQ. However, these three types of PPOs were not enriched in the association analysis model because PPOs expression levels were high in all strains and there was no significant difference. This led to the omission of PPOs based on differential gene association analysis. In this study, PPOs were significantly enriched in GSEA analysis, and PPOs expression in purple strains began to increase significantly during the primordium stage, coinciding with pigment secretion. Therefore, it is inferred that PPOs are the key enzymes involved in pigment synthesis. The RT qPCR results showed that there was no significant difference in the expression levels of the three PPOs among different colored strains, but the expression levels were high (Figure 6), which is consistent with our analysis results. It is currently unknown whether all three types of PPOs are involved in pigment synthesis, research has shown that PPOs not only regulate pigment synthesis, but also participate in defense mechanisms against stress (Boss et al., 1995), which may be the reason for the high expression levels of all three types of PPOs.

## Materials and methods

### Test strains

The test strains were wild white strain ACW001 and wild purple strain ACP004, both collected from Henan Province, China. ACW001-33 was the monospore collected from ACW001 and used for whole-genome sequencing. The above strains were stored at the Engineering Research Center for Edible and Medicinal Fungi of Jilin Agricultural University, China.

### DNA preparation and sequencing

As the test strain, ACW001-33 was extracted using CTAB and stored at −80°C for future use. Sequencing was performed using the CLR and CCS libraries. The one with better results and accuracy was finally selected. 20 Kb SMRT Bell library and 350 bp small fragment library were constructed on PacBio Sequel and Illumina NovaSeq PE150. After the library test, different libraries were sequenced according to the effective concentration and the amount of output data. Genome assembly and correction of reads were performed with SMRT Link v5.0.1 (Ardui et al., 2018). The reads were aligned to the assembled genome sequence, and the GC bias of the genome was summarized by computing the GC content and coverage depth of the reads in the assembled sequence.

### Hi-C-assisted assembly

Samples were first cross-linked using formaldehyde for library sequencing. Following quality control and filtration of hic tags with HiCUP, valid reads for interaction analysis were aligned to the reference genome (Lieberman-Aiden et al., 2009). The number of reads with interactions between contigs was counted, which was also considered the number of interactions. Contigs were clustered according to this number, and sequencing and orientation were then performed based on the interaction intensity of every two contigs and the position of the interacting read alignment. Genome sequences were clustered, ordered, and oriented with ALLHiC, and contig orientation was corrected with hic-hicker (Ryo and Shinichi, 2020).

### Genomic component analysis

#### Repetitive sequence

Interspersed repeat sequences were predicted using Repeat Masker, and tandem repeat sequences were searched by TRF (Tandem repeats finder) (Benson, 1999). An SSR search was used for detecting genome-wide SSR loci and designing primers.

#### Encoding gene

Augustus prediction was performed *because* the *A. cornea* genome has no transcriptome data or adjacent reference sequence (Stanke et al., 2008). Based on the final assembly result (≥500 bp), ORF (Open Reading Frame) prediction and filtration were performed.

#### Noncoding RNA

tRNA prediction was performed using tRNAscan-SE (Lowe and Eddy, 1997). rRNA was predicted using rRNAmmer (Lagesen et al., 2007). Predictions of sRNA, snRNA, and miRNA were similar.

### Gene function analysis

Regarding coding sequences, we performed functional annotations on different databases, including frequently used KEGG, KOG, Pfam, CAZy, NR, GO, Swiss-Prot, and TCDB, as well as a pathogenicity-specific database. BLAST was used to compare putative genes with each functional database. For the BLAST results of each sequence, the comparison result with the highest score was selected (default: identity ≥40%, coverage ≥40%) for annotation. We also analyzed effectors, including secretory protein, cellular pigment P450, and secondary metabolic gene clusters. A signal peptide prediction tool SignalP was used to predict secretory protein, detect the presence of signal peptides and transmembrane results, and predict whether the protein sequence was secreting protein (Petersen et al., 2011). Annotation was performed using the cytochrome database P450. Predictive analysis of secondary metabolic gene clusters was performed using antiSMASH (Medema et al., 2011).

### Phylogenetic development, evolutionary, and whole-genome collinearity analysis

OrthoMCL (v.2.0.9) (Li et al., 2003) was used to identify homologous gene families based on the genome sequences and protein files of 20 species, including *A. cornea*, *A. heimuer*, and *A. subglabra* downloaded from NCBI. Shared single-copy genes were selected and aligned using Clustal Omega (Sievers and Higgins, 2018). A genome-based phylogenetic tree was constructed using the maximum likelihood (ML) algorithm in RAxML (Stamatakis, 2014). Computational Analysis of Gene Family Evolution (CAFE) 3.1 (De Bie et al., 2006) was used to predict the expansion and contraction of homologous gene families, and a value of *p* of <0.05 was considered significant. Positively selected genes were determined using the branch-site model in the CodeML tool of PAML (Gao et al., 2019). To determine large-scale collinearity relationships between genomes, the MUMmer software (Version 3.23) was used to align the target and reference genomes. LASTZ (Version 1.03.54) was then used to confirm local positional arrangements and search for regions of translocation (Translocation/Trans), inversion (Inversion/Inv), and translocation+inversion (Trans+Inv).

### Transcriptomic analysis of different color strains

Data from three different development stages of the white strain ACW001 and purple strain ACP004, including the mycelium period, primordium period (8 days), and fruiting body period (15 days), were collected for total RNA extraction and sequencing. Raw reads were first subjected to quality control with fastp to exclude poor-quality data (Chen et al., 2018). Clean reads were then obtained, which were aligned to the ACW001-33 genome by HISAT2 (Kim et al., 2015). Based on the alignment results, we reconstructed a transcript using Stringtie and calculated all gene expression levels in each sample using RSEM (Pertea et al., 2015). The expression level was displayed with raw reads count and FPKM for subsequent analysis of differences between samples and groups. Finally, by screening the significantly different genes, the selected genes were analyzed by KEGG and GO enrichment to identify pigment synthesis-related pathways and key pigment synthesis-responsible genes (Love et al., 2014).

### Metabonomic analysis of different color strains

The fruiting bodies of ACW001 and ACP004 were collected for nontargeted LC–MS metabolite detection. Both positive and negative ion modes were used in the subsequent data analysis during detection. Multivariate data analysis was performed using R language gmodels (v2.18.1) for PCA analysis and the ropls package for OPLS-DA analysis (Warnes, 2007; Julien and Douglas, 2013). We combined the VIP value of OPLS-DA and *p*-value of the T-test to screen DEMs between comparison groups (Saccenti et al., 2014). Finally, we performed KEGG enrichment and topology analyses of DEMs.

## Data availability statement

The datasets presented in this study can be found in online repositories. The names of the repository/repositories and accession number(s) can be found at: https://www.ncbi.nlm.nih.gov/, PRJNA943604, https://www.ncbi.nlm.nih.gov/, PRJNA944815.

## Author contributions

PW and FY: conceptualization. PW and XM: methodology and formal analysis. MF: software and data curation. XS, LL, and MF: validation. XM and FY: investigation. FY: resources, project administration, and funding acquisition. XM: writing—original draft preparation and visualization. YZ: writing—review and editing. KS, LL, and FY: supervision. All authors have read and agreed to the published version of the manuscript.

## Funding

This work was supported in part by funds from the China Agriculture Research System, grant number CARS-20 and the Guizhou Key Laboratory of Edible fungi breeding (No. [2019]5105-2001 and No. [2019]5105–2005).

## Conflict of interest

The authors declare that the research was conducted in the absence of any commercial or financial relationships that could be construed as a potential conflict of interest.

## Publisher’s note

All claims expressed in this article are solely those of the authors and do not necessarily represent those of their affiliated organizations, or those of the publisher, the editors and the reviewers. Any product that may be evaluated in this article, or claim that may be made by its manufacturer, is not guaranteed or endorsed by the publisher.
