# Supplementary material for: High-quality genome assembly and multi-omics analysis of pigment synthesis pathway in Auricularia cornea
Source: Front Microbiol. 2023 Jun 15;14:1211795. doi: 10.3389/fmicb.2023.1211795 (PMC10308021; doi:10.3389/fmicb.2023.1211795)
Supplement: Supplementary file 1 [file Data_Sheet_1.docx]

Supplementary Material

High-quality genome assembly and multi-omics analysis of pigment synthesis pathway in *Auricularia cornea*

Xiaoxu Ma^1,2†^, Lixin Lu^3†^, Fangjie Yao^1,3,*^, Ming Fang^3^, Peng Wang^4^, Jingjing Meng^3^, Kaisheng Shao^1^, Xu Sun^1^, Youmin Zhang^3^

*** Correspondence:** yaofj@aliyun.com (F.Y.)

# Supplementary Figures and Tables

## Supplementary Figures

#
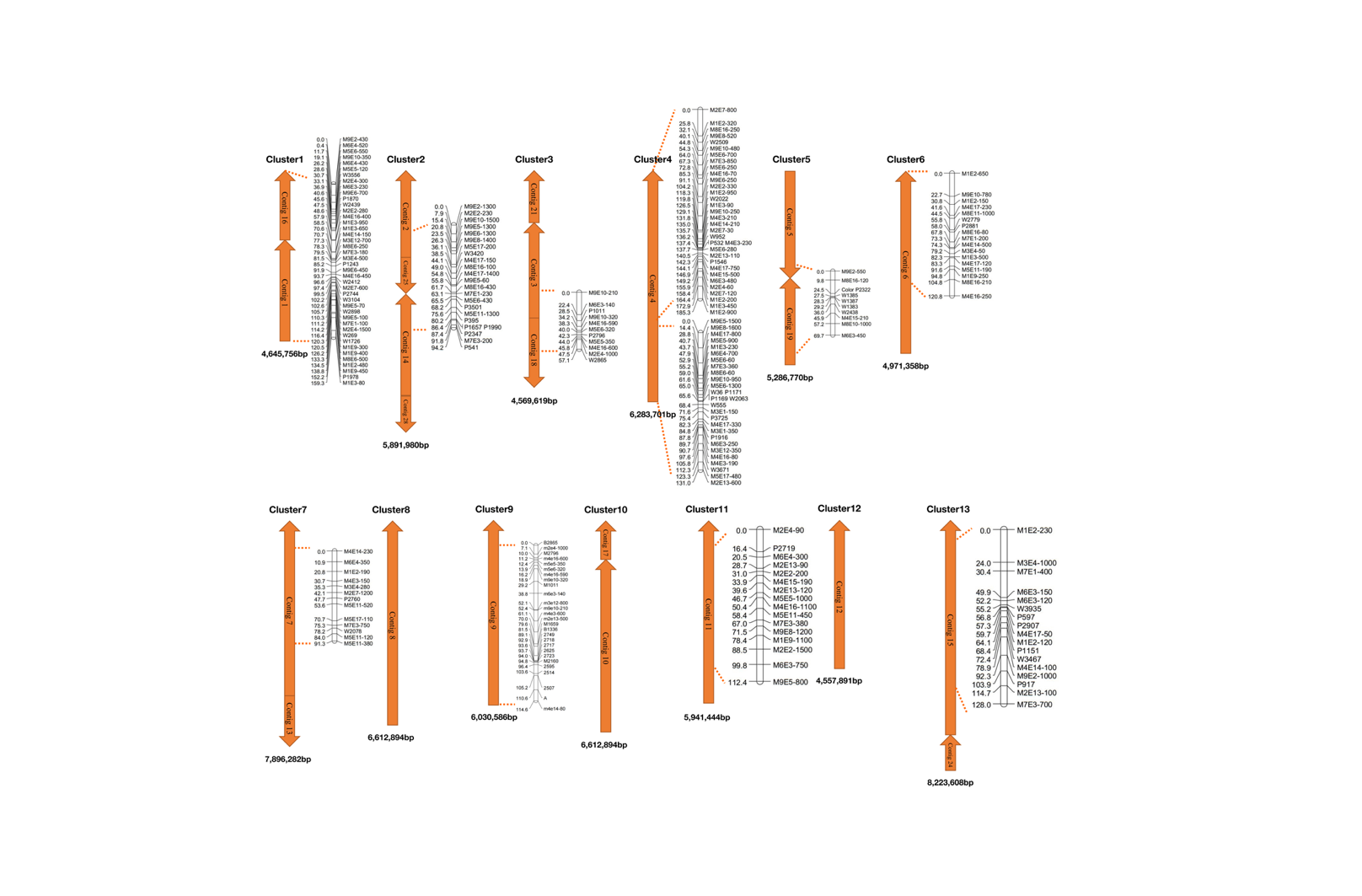


**Supplementary Figure 1.** **Collinearity analysis of genetic linkage and physical maps.** The arrows represent the direction of contigs, and the genetic linkage map is anchored at the corresponding position of each cluster after sequence alignment.

## Supplementary Tables

Supplementary Table S1. POS ion mode difference metabolite table

| **Index** | **log2_FC** | **Pvalue** | **FDR** | **VIP** |
| --- | --- | --- | --- | --- |
| Com_1_pos | 0.15496821 | 0.0124972 | 0.01947137 | 11.9965799 |
| Com_19_pos | -5.0924655 | 2.91E-07 | 1.75E-06 | 9.68168475 |
| Com_23_pos | 1.33096903 | 3.82E-10 | 1.95E-08 | 6.8030046 |
| Com_24_pos | 1.32961876 | 3.82E-10 | 1.95E-08 | 6.79367729 |
| Com_20_pos | -1.1652949 | 2.98E-06 | 1.22E-05 | 6.68205991 |
| Com_43_pos | 3.60861474 | 6.63E-10 | 2.08E-08 | 6.58190097 |
| Com_17_pos | 0.51525606 | 2.18E-06 | 9.38E-06 | 6.21989841 |
| Com_40_pos | 1.36035141 | 1.42E-09 | 2.89E-08 | 5.37281921 |
| Com_18_pos | -0.3589544 | 0.00027364 | 0.00061654 | 4.68415173 |
| Com_9_pos | 0.16725543 | 0.02497051 | 0.0368008 | 4.03086645 |
| Com_27_pos | 0.41929829 | 0.0004979 | 0.00104827 | 3.85335929 |
| Com_48_pos | 0.66543079 | 3.38E-05 | 9.77E-05 | 3.66896407 |
| Com_82_pos | 1.22809092 | 8.36E-10 | 2.21E-08 | 3.57076096 |
| Com_86_pos | 1.23703615 | 7.29E-10 | 2.08E-08 | 3.54825253 |
| Com_105_pos | -1.9329518 | 1.18E-09 | 2.65E-08 | 3.37331433 |
| Com_147_pos | 2.30403123 | 7.56E-08 | 5.44E-07 | 3.24047869 |
| Com_80_pos | -1.2053704 | 5.24E-05 | 0.00014022 | 3.08268242 |
| Com_74_pos | 1.19884072 | 0.00224783 | 0.00411568 | 3.05719507 |
| Com_114_pos | 1.03912009 | 1.49E-08 | 1.52E-07 | 2.87522721 |
| Com_76_pos | -3.089786 | 0.00912138 | 0.01466927 | 2.81302131 |
| Com_115_pos | 0.96205099 | 5.52E-05 | 0.00014631 | 2.60992461 |
| Com_58_pos | 0.7431503 | 0.01219672 | 0.0190362 | 2.60814044 |
| Com_262_pos | 8.73765399 | 1.43E-08 | 1.50E-07 | 2.51545783 |
| Com_96_pos | 0.58358105 | 8.17E-05 | 0.00020504 | 2.44957414 |
| Com_135_pos | 0.8827425 | 5.79E-07 | 3.08E-06 | 2.41962893 |
| Com_200_pos | -1.9195904 | 1.76E-09 | 3.39E-08 | 2.31045585 |
| Com_30_pos | -0.2194794 | 0.03068302 | 0.04420518 | 2.22432591 |
| Com_178_pos | 0.91878591 | 3.43E-07 | 2.02E-06 | 2.16555659 |
| Com_204_pos | -1.3282632 | 4.43E-07 | 2.52E-06 | 2.0183449 |
| Com_151_pos | 0.57152487 | 8.52E-06 | 2.99E-05 | 1.98841405 |
| Com_319_pos | -3.7940517 | 1.37E-13 | 1.23E-10 | 1.92816056 |
| Com_65_pos | 0.27602878 | 0.00314118 | 0.0055589 | 1.85334753 |
| Com_64_pos | -0.3135434 | 0.00764966 | 0.0126649 | 1.83308401 |
| Com_72_pos | -0.309292 | 0.00667062 | 0.01114663 | 1.83059815 |
| Com_233_pos | 0.80547895 | 1.41E-07 | 9.38E-07 | 1.7520963 |
| Com_376_pos | 1.76749383 | 1.05E-09 | 2.56E-08 | 1.7411742 |
| Com_287_pos | -2.2370227 | 5.20E-07 | 2.88E-06 | 1.73542715 |
| Com_324_pos | 1.45098913 | 2.64E-05 | 7.88E-05 | 1.69448861 |
| Com_337_pos | 1.33041765 | 8.04E-09 | 1.02E-07 | 1.60544809 |
| Com_454_pos | -3.6163243 | 6.97E-10 | 2.08E-08 | 1.59180792 |
| Com_346_pos | -2.2519166 | 0.00042933 | 0.00091461 | 1.54972897 |
| Com_289_pos | -0.9820494 | 9.19E-06 | 3.16E-05 | 1.48189309 |
| Com_145_pos | -0.3810401 | 0.00160444 | 0.00302388 | 1.47519187 |
| Com_499_pos | -2.7300725 | 5.60E-10 | 2.08E-08 | 1.47250119 |
| Com_166_pos | -0.4692059 | 0.00190575 | 0.00354715 | 1.4510759 |
| Com_312_pos | -1.1701225 | 2.84E-05 | 8.43E-05 | 1.4411389 |
| Com_628_pos | 3.8159316 | 5.11E-08 | 4.11E-07 | 1.41837457 |
| Com_373_pos | 1.25727192 | 6.92E-05 | 0.00017886 | 1.38404616 |
| Com_183_pos | 0.59450062 | 0.00825082 | 0.01348633 | 1.3802272 |
| Com_97_pos | -0.2135281 | 0.00815343 | 0.01337579 | 1.34616134 |
| Com_238_pos | 0.46718699 | 7.98E-05 | 0.00020212 | 1.33181297 |
| Com_594_pos | 5.46649823 | 0.00018376 | 0.0004274 | 1.32369426 |
| Com_639_pos | 3.34534679 | 2.72E-10 | 1.63E-08 | 1.31436077 |
| Com_547_pos | -1.9999148 | 4.20E-09 | 5.90E-08 | 1.2905119 |
| Com_510_pos | -1.6125702 | 6.23E-08 | 4.83E-07 | 1.28575616 |
| Com_599_pos | -2.5003738 | 2.10E-10 | 1.63E-08 | 1.27016445 |
| Com_99_pos | 0.20987565 | 0.02718809 | 0.03967872 | 1.26076526 |
| Com_146_pos | -0.3245872 | 0.01803575 | 0.02706867 | 1.25606387 |
| Com_405_pos | -1.2058446 | 3.20E-06 | 1.29E-05 | 1.2238553 |
| Com_314_pos | -0.8536318 | 0.00031484 | 0.00069543 | 1.2122204 |
| Com_296_pos | -0.5961615 | 2.34E-05 | 7.15E-05 | 1.20374893 |
| Com_568_pos | -2.6867044 | 1.24E-08 | 1.41E-07 | 1.19949926 |
| Com_104_pos | 0.20716667 | 0.02712091 | 0.03967872 | 1.19570416 |
| Com_582_pos | -1.8711937 | 5.65E-08 | 4.46E-07 | 1.18495809 |
| Com_311_pos | 0.51864882 | 3.09E-05 | 9.03E-05 | 1.1833146 |
| Com_562_pos | -5.2227153 | 6.69E-06 | 2.38E-05 | 1.17161629 |
| Com_283_pos | -0.6436961 | 0.00839016 | 0.01368921 | 1.16792883 |
| Com_122_pos | 0.22147494 | 0.01992535 | 0.0296571 | 1.15609745 |
| Com_644_pos | -1.827259 | 5.11E-08 | 4.11E-07 | 1.14699561 |
| Com_664_pos | -1.8610776 | 2.38E-08 | 2.23E-07 | 1.09294869 |
| Com_173_pos | -0.5448004 | 0.04660434 | 0.06475626 | 1.08071354 |
| Com_945_pos | -4.5700988 | 9.22E-10 | 2.37E-08 | 1.08060263 |
| Com_678_pos | -4.329701 | 6.99E-07 | 3.51E-06 | 1.08004491 |
| Com_320_pos | -0.5145948 | 8.89E-05 | 0.00022196 | 1.07183453 |
| Com_771_pos | -2.6044747 | 1.18E-07 | 8.03E-07 | 1.06752264 |
| Com_442_pos | -0.7667965 | 1.03E-05 | 3.50E-05 | 1.05143615 |
| Com_901_pos | 2.06379083 | 2.97E-09 | 4.95E-08 | 1.03617969 |
| Com_642_pos | 0.9573923 | 1.25E-06 | 5.78E-06 | 1.02782176 |
| Com_719_pos | -1.6304299 | 1.11E-09 | 2.63E-08 | 1.01199804 |
| Com_935_pos | -2.8231309 | 1.17E-09 | 2.65E-08 | 1.00981811 |
| Com_391_pos | 0.44218625 | 5.76E-06 | 2.09E-05 | 1.00187996 |

Supplementary Table S2. NEG ion mode difference metabolite table

| **Index** | **log2_FC** | **Pvalue** | **FDR** | **VIP** |
| --- | --- | --- | --- | --- |
| Com_1_neg | 0.37518258 | 0.02822235 | 0.03895555 | 9.92642793 |
| Com_10_neg | 0.85115118 | 0.00107859 | 0.00214817 | 7.75719551 |
| Com_18_neg | 0.96696435 | 0.00767459 | 0.01222123 | 6.18332409 |
| Com_6_neg | 0.21935309 | 0.0025276 | 0.00455223 | 5.71137068 |
| Com_24_neg | 0.82487932 | 0.00597947 | 0.00976101 | 5.4159538 |
| Com_21_neg | -0.9830009 | 0.00088126 | 0.00180029 | 5.41131268 |
| Com_56_neg | 2.49935227 | 7.10E-06 | 2.64E-05 | 5.19764476 |
| Com_70_neg | 1.38615177 | 9.69E-09 | 1.02E-07 | 4.60518535 |
| Com_39_neg | 0.78618009 | 7.25E-06 | 2.68E-05 | 4.52215653 |
| Com_57_neg | 0.96514584 | 9.68E-06 | 3.43E-05 | 4.14973298 |
| Com_85_neg | 2.2640935 | 1.65E-07 | 1.20E-06 | 4.08456834 |
| Com_49_neg | 0.76689741 | 0.00220573 | 0.00406468 | 3.66052075 |
| Com_72_neg | 1.04694379 | 0.00047978 | 0.00104268 | 3.43661488 |
| Com_94_neg | 0.92833241 | 6.39E-09 | 7.40E-08 | 3.24798516 |
| Com_68_neg | 0.57295517 | 0.00011977 | 0.00030047 | 3.10843588 |
| Com_101_neg | -1.2423401 | 7.87E-11 | 2.56E-09 | 3.04006904 |
| Com_136_neg | -2.768936 | 1.98E-07 | 1.39E-06 | 2.883997 |
| Com_97_neg | 0.67739775 | 2.46E-07 | 1.66E-06 | 2.82396224 |
| Com_98_neg | -2.8096519 | 0.00480346 | 0.00806214 | 2.69798174 |
| Com_79_neg | -0.5759086 | 3.97E-06 | 1.62E-05 | 2.59924462 |
| Com_77_neg | -0.5328087 | 8.67E-06 | 3.15E-05 | 2.58529778 |
| Com_71_neg | -0.4104572 | 3.79E-05 | 0.00011103 | 2.45654134 |
| Com_138_neg | -0.9883893 | 1.30E-09 | 1.93E-08 | 2.44310726 |
| Com_120_neg | -0.8083603 | 7.39E-09 | 8.13E-08 | 2.40986284 |
| Com_145_neg | 0.72521668 | 1.32E-05 | 4.40E-05 | 2.21255456 |
| Com_134_neg | 0.54602528 | 2.53E-07 | 1.69E-06 | 2.16712237 |
| Com_86_neg | 0.5324621 | 0.00853172 | 0.01334832 | 2.14797271 |
| Com_306_neg | 1.5878906 | 7.85E-11 | 2.56E-09 | 2.04021878 |
| Com_484_neg | 8.18157477 | 1.10E-12 | 1.97E-10 | 1.96729975 |
| Com_362_neg | 1.62005048 | 5.11E-09 | 6.31E-08 | 1.89194005 |
| Com_197_neg | 0.77630983 | 0.00085472 | 0.00175107 | 1.81791795 |
| Com_621_neg | 6.05318269 | 7.04E-13 | 1.97E-10 | 1.77855126 |
| Com_468_neg | 2.11792963 | 1.69E-08 | 1.68E-07 | 1.77155596 |
| Com_222_neg | 1.19845001 | 0.00183793 | 0.00345821 | 1.73947677 |
| Com_230_neg | 0.69625955 | 2.75E-06 | 1.22E-05 | 1.70439535 |
| Com_387_neg | -1.5186832 | 7.39E-10 | 1.23E-08 | 1.63593268 |
| Com_186_neg | 0.42591531 | 7.97E-07 | 4.25E-06 | 1.60769517 |
| Com_415_neg | -1.4702844 | 1.79E-10 | 4.12E-09 | 1.59568256 |
| Com_141_neg | -0.5884134 | 0.01320495 | 0.01979358 | 1.57375459 |
| Com_179_neg | 0.65635326 | 0.02307479 | 0.0324135 | 1.56966053 |
| Com_246_neg | -0.9549415 | 0.00182072 | 0.00343488 | 1.49749068 |
| Com_512_neg | 2.05913094 | 4.68E-06 | 1.85E-05 | 1.46737437 |
| Com_360_neg | 0.85325127 | 3.41E-05 | 0.00010188 | 1.41487402 |
| Com_187_neg | 0.52220348 | 0.00950782 | 0.0146827 | 1.4142948 |
| Com_168_neg | -0.6576637 | 0.02602792 | 0.03620616 | 1.40128063 |
| Com_565_neg | -2.1036437 | 7.23E-09 | 8.08E-08 | 1.39800092 |
| Com_372_neg | 0.73552718 | 3.85E-10 | 7.40E-09 | 1.39394236 |
| Com_91_neg | 0.21362959 | 0.01720831 | 0.02485645 | 1.37534527 |
| Com_359_neg | 0.88459043 | 0.00062882 | 0.00133019 | 1.35518957 |
| Com_437_neg | -1.0736783 | 3.96E-06 | 1.62E-05 | 1.28544432 |
| Com_289_neg | 0.8365753 | 0.00824313 | 0.01295349 | 1.27150391 |
| Com_554_neg | -1.8996742 | 9.67E-07 | 4.94E-06 | 1.24603825 |
| Com_767_neg | 1.7823044 | 2.22E-06 | 1.02E-05 | 1.24041975 |
| Com_314_neg | 0.51373172 | 0.00011261 | 0.00028551 | 1.20844746 |
| Com_513_neg | 0.77234954 | 2.92E-06 | 1.26E-05 | 1.20437628 |
| Com_923_neg | -2.8083638 | 5.01E-11 | 2.24E-09 | 1.17850343 |
| Com_763_neg | -1.9385844 | 2.33E-10 | 5.05E-09 | 1.16665088 |
| Com_305_neg | 0.61500154 | 0.01869861 | 0.02666834 | 1.13981817 |
| Com_228_neg | 0.40680976 | 0.0056254 | 0.00926765 | 1.11609553 |
| Com_602_neg | 0.98741309 | 0.00013631 | 0.00033264 | 1.10500023 |
| Com_949_neg | 6.83067161 | 2.69E-05 | 8.24E-05 | 1.08599748 |
| Com_215_neg | -0.2906483 | 0.00050243 | 0.00108531 | 1.07713442 |
| Com_936_neg | -1.9180052 | 1.56E-10 | 3.72E-09 | 1.07100617 |
| Com_563_neg | -0.9239324 | 0.00011788 | 0.00029677 | 1.06803451 |
| Com_1503_neg | 7.07016564 | 2.44E-11 | 1.94E-09 | 1.05635531 |
| Com_818_neg | 0.96132339 | 7.51E-08 | 5.97E-07 | 1.04556354 |
| Com_617_neg | 0.78170361 | 7.58E-05 | 0.00019984 | 1.03857686 |
| Com_652_neg | 0.65944284 | 2.97E-07 | 1.95E-06 | 1.01300708 |
| Com_591_neg | 1.40189492 | 0.01614487 | 0.0234657 | 1.00529253 |

Supplementary Table S3. 20 RT-qPCR primers related to pigment synthesis

| **Gene ID** | **Left Primer** | **TM(℃)** | **Right Primer** | **TM(℃)** |
| --- | --- | --- | --- | --- |
| A01041 | GGTGACGGCGCTACTCTACG | 61 | AAGCCGTGTTGCGACATCTTC | 60 |
| A03098 | CGTACACCAGTTCATCCTCCAGC | 61 | CGTCCTTCTCGAGCTCGTGG | 61 |
| A03226 | GACATAGTTAAGCCTGGCGTCC | 60 | CTGCTGCTGGTCGTTGTTGAG | 60 |
| A12464 | GTTGACCTCTGGACGTCCAAGG | 61 | TTCCTTCTGCTTCGTGACGGC | 61 |
| A14758 | TGAACATCGTGCGGATGAACT | 60 | CATCCACGTAGACGAGCTTGC | 60 |
| A14911 | GCTGGCGACGTTATTCTCGAGAT | 61 | CGTCCTTCTTCTTAGGCTGGGAC | 61 |
| A10447 | TACCATTGGTCTTGCCAAGTGG | 60 | CGCACCGTGTTGATCTCACC | 60 |
| A01610 | TACTACCTCGTCGCATACAACGT | 60 | GACTGGACGACGGTCGTCTG | 60 |
| A06202 | GATGGACTCGCTCAAGCGC | 60 | CCTTGAGACTCTCGACGACGC | 61 |
| A02162 | TCATCTGGCAGAAGATTCGCG | 60 | CGTTGGGCGACTTCCACATG | 60 |
| A00253 | GAGCTCAGCCACTACGGCTG | 61 | ATGAGTCGGTCGAGGATCATGC | 61 |
| A05967 | GCAGATGGCAGAGTCCATCTCG | 61 | CTGCTTCTGCTGCTCCTGC | 61 |
| A05323 | GTGGAATTGGCCAGCCGTTAT | 60 | CGAGATGATGAGCAGGTGCG | 60 |
| A08004 | GCTCATTGAGCACGAGTACGAC | 60 | TGACGGTCTTAGGTGCTTCTCTG | 60 |
| A05690 | CTACGTTCAAGCCGGAGAGC | 60 | CAGCTTGACCAAGACCAGAAGC | 60 |
| A12376 | GGATGGAGAATTCCGCTTCGG | 61 | GGTTCTCGCTCGTGATGTTGG | 60 |
| A01747 | TGGCACAGAGGCTTCATCTCG | 61 | CGTATTGGTTCAGGTCGCGC | 60 |
| A03568 | CACGGCAAGAACCTCATCGG | 60 | GAGGATTATGCCGACCGAGACG | 61 |
| A10754 | GGCGGTAGTATGCGAGTTGC | 61 | AATTGCCGTGCCATTCGACG | 61 |
| A02009 | CCGGTCGTCGTGTTAGTCAAG | 60 | GGACGGCCTTGACATTCGT | 60 |
